# Belgian Radiology Research in Full Power!

**DOI:** 10.5334/jbr-btr.1240

**Published:** 2017-02-27

**Authors:** Piet Vanhoenacker

**Affiliations:** 1 OLVZ Aalst, BE

**Keywords:** Radiology, Research, Thesis, Belgium

## Abstract

In Belgium, there has always been a strong tradition to foster radiology and imaging research. The former Royal Belgian Society of Radiology (RBSR-KBVR-SRBR)—after the merger with the Consilium and NUR, now the Belgian Society of Radiology (BSR-BVR-SBR)—has persistently been one of the driving forces of support to individuals who wanted to innovate and put Belgian radiology research at the forefront.

At the current symposium, we wanted to present and acknowledge the hard work of our Belgian colleagues who have obtained a doctoral degree related to imaging and radiology during the years 2015–2016.

We have chosen to give these colleagues the opportunity to present their curriculum and work as a review article in the current printed symposium issue of the *JBSR*, the e-journal that is the newer version of the former, well-known *JBR-BTR*. Thanks to the efforts of the authors and Editor-in-Chief Dr. Alain Nchimi, we are able to give an update on the scientific doctoral work being done in Belgium.

The following is a short presentation of the CV of the colleagues who have been financially supported, through the doctoral thesis program.

A synopsis of their actual work is published in this symposium issue of the *JBSR*.

**Dr. Alain Nchimi (2015)**


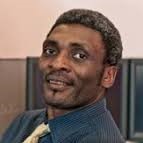


Dr. Alain Nchimi is currently the Editor-in-Chief of the *Journal of the Belgian Society of Radiology* and a body imaging and paediatric radiologist in the medical imaging departments of the Centre Hospitalier du Luxembourg and the Children’s University Hospital Queen Fabiola of Brussels. He is a member of the Belgian Society of Radiology and chair of the Cardiovascular Imaging Section. He earned his medical degree and completed a master’s degree in medical imaging at the University of Liège in 2002. In 2010, he started a research program in the “GIGA cardiovascular diseases” translational research platform of Liège University, with the aim of finding new imaging biomarkers for various cardiovascular diseases, including heart valve diseases, aortic dissection, and aneurysms. His research on abdominal aortic aneurysms (AAA) was supported by a collaborative grant of the 7th FP European Framework Program (Fighting Aneurysmal Disease) and a grant of the BSR. He obtained a PhD from the University of Liège in 2015 for an original dissertation titled “Imaging the mechanisms involved in abdominal aortic aneurysms rupture; a step towards patient-specific risk assessment”, for which a summary is published in the current issue of the JBSR as a review article under the title “Cross-sectional imaging to evaluate the risk of rupture in Abdominal Aortic Aneurysms” [1].

The clinical part of his work aimed to determine how far imaging biological activities may help clinical decision making in patients with AAA and its incremental value, as compared to the diameter-based patient management algorithm, whereas the experimental part looked at new and promising imaging concepts for the assessment of biological processes in AAA. The main findings of his works are the following: (i) Increased 2F-fluorodeoxyglucose (FDG) uptake on positron emission tomography (PET) was a diameter-independent marker of AAA-related events over two years. (ii) Part of the FDG uptake is associated with biological activities along the luminal surface of the ILT, where phagocytosis of superparamagnetic iron oxide was shown on MRI, both ex vivo and in vivo. (iii) This phagocytosis was correlated with the abundance of leukocytes and proteolytic activity. (iv)The MRI appearances of the intraluminal thrombus (ILT) resulted from the endogenous iron distribution related to the same biological activities. (v) Multimodality imaging was used to proof the concept of the deleterious role of the ILT in AAA growth in a rat model of AAA.

In summary, the research done by Dr. Nchimi shows that MRI and FDG PET are both capable of evidencing and quantifying in vivo some of the notoriously deleterious biological processes taking place in the aneurysmal sac, especially those related to the entrapped phagocytes and red blood cells in the ILT and the periadventitial inflammatory response.

**Dr. Nele Herregods (2016)**


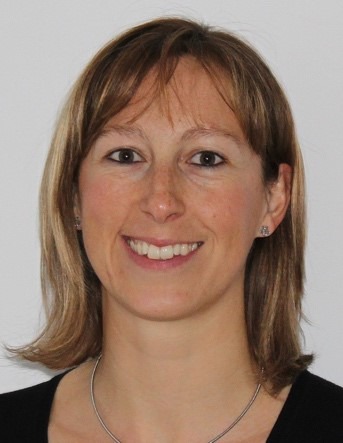


Dr. Nele Herregods obtained her MD at Ghent University in 2004 at the University of Ghent, Belgium. She received a PhD in 2016 at the same institution, with her doctoral thesis on MRI of the sacroiliac joints in children with spondyloarthritis, of which a summary is published in the current issue of the *JBSR* as a pictorial review article under the title “Diagnostic value of MRI of the sacroiliac joints in juvenile spondyloarthritis” [2]. She is currently working as head of clinic in the Department of Radiology at the Princess Elisabeth Children’s Hospital at Ghent University Hospital.

Her research work aimed to determine the diagnostic value and potential role of sacroiliac joint MRI in juvenile spondyloarthritis. The main findings of the works are the following: (i) There are multiple features of active inflammation and structural damage visible on MRI of the sacroiliac joints that can provide a specific diagnosis of JSpA when present in children with suspected sacroiliitis. In many cases, different features are seen concomitantly, making it easier to diagnose sacroiliitis with more confidence. (ii) MRI without contrast administration is sufficient to identify bone marrow edema, capsulitis, and retro-articular enthesitis as features of active sacroiliitis in JSpA. Only in selected cases, gadolinium-enhanced images may help to confirm the presence of synovitis. (iii) The adult ASAS definition for a “positive” MRI needs some adaptations for children. (iv) There is a high correlation between pelvic enthesitis and sacroiliitis on MRI of the sacroiliac joints in children. As pelvic enthesitis indicates active inflammation, it may play a role in assessment of the inflammatory status.

In summary, the research done by Dr. Herregods shows that MRI of the sacroiliac joints in children is a useful tool and should be applied in clinical practice in children suspected for JSpA.

**Dr. Erik Ranschaert (2016)**


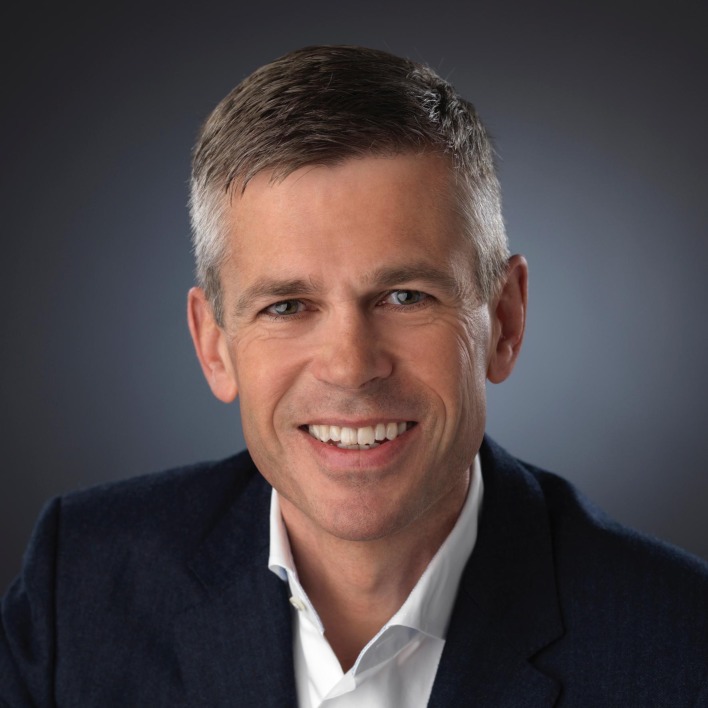


Dr. Erik Ranschaert is currently active at the Radiology Department of the University Hospitals in Leuven. His main field of interest is abdominal and urogenital imaging. As of January 2017, he will be chief of the Radiology Department at the Heilig Hartziekenhuis in Mol, Belgium. He graduated in radiology in 1994 at the University Hospitals of Leuven. The start of his radiological activities on the Internet in the late 1990s were pivotal for his later fascination and dedication for informatisation and digitisation of radiology, including teleradiology. He obtained his PhD in 2016 at the University Hospital of Antwerp for a dissertation titled “The impact of information technology on radiology services” [3].

He is an active member of the Belgian Society of Radiology (BSR), the European Society of Radiology (ESR), the European Society of Gastro-abdominal Radiology (ESGAR), the ESOI (European Society of Oncologic Imaging), the Radiological Society of North America (RSNA), and the Society for Imaging Informatics in Medicine (SIIM). He was active as a member and chairman of the ECR Computer Applications subcommittee from 2006 till 2008. He was a member of the ICT-working group of the NVvR (Radiological Society of the Netherlands, 2012–2014) and was a member of the ESR e-Health and Informatics subcommittee (2013–2016). During that period, he was member of the RSNA/ESR Template Library Panel (TLAP) for Structured Reporting. In 2016, he was elected as vice president of the European Society of Medical Imaging Informatics (EuSoMII).

He was an editorial board member of the scientific journal *Insights into Imaging* (2009–2013) and is a reviewer for several journals. Between 1998 and 2011, he was an editorial board member of the radiological magazine *Diagnostic Imaging Europe*, for which he published 20 columns. Between the years 2009 and 2012, he was co-editor of Radiopaedia.org, where he published 46 cases and edited several articles in the field of abdominal imaging. He gave more than 40 lectures at national and international courses and conferences on topics related to information technology and teleradiology. He authored and co-authored 18 peer-reviewed articles, of which 12 are on the subject of this thesis, and he published two thesis-related scientific posters on the ESR Electronic Presentation Online System (EPOS).

In his dissertation, the ongoing revolution in medicine is described, which is mainly caused by the rapid evolution of the Internet and the concomitant exponential progress in the digitisation of medical information. The main goal of his work was to demonstrate the following: (i) Both the digitisation of medical imaging and the continuous progress in information and communication technology have had and still have a significant impact on the way by which radiological services are and can be delivered. (ii) The possibility to exchange medical information and radiological images digitally has played a crucial role in the establishment of teleradiology. (iii) Teleradiology is here to stay, although chances are high that the format and types of applications of teleradiology will change, as both the underlying technology and the practice of medicine are currently subject to crucial changes. (iv) Major new trends, such as the increasing popularity of mobile devices and social media, the progress of e-Health as part of the Internet of Things, and growing patient empowerment, will have a significant impact on the way radiological services are delivered in the future.

Based upon his analysis and work, he concludes that the future radiologist will play a central role in a more personalised imaging-guided process of diagnosis and treatment. The role of the radiologist as manager and service provider is redefined in a more patient-centric model, in which information technology has an indispensable role. The way radiologists deal with artificial intelligence (AI) is crucial in this context: AI should rather be considered as a form of intelligence amplification (IA), a technique able to reinforce the importance and usefulness of diagnostic imaging.

**Dr. Johan Coolen (2015)**


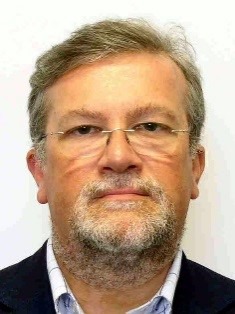


Dr. Johan Coolen is currently working as a chest radiologist in the Department of Radiology at the University Hospitals of Leuven. He earned his MD and completed a master’s degree in medical imaging at the Catholic University of Leuven in 1991 and joined the chest team in 2008. Initially, he focused on the quantification of COPD diagnosis, but after obtaining a grant from the Foundation Against Cancer, his natural impulse to research became obvious. Moreover, different travel awards during congresses of International Mesothelioma Imaging Group, European Society of Thoracic Imaging, Radiological Society of North America, and European Respiratory Society (ERS) gave him the opportunity to become chair (2011–2014) and secretary (2014–2017) of the Imaging Group 3.1 of the ERS.

The focus of his research program shifted to thoracic MR imaging and aimed to implement innovative MR research in clinical situations, leading in 2015 to a successful dissertation titled “Diffusion- and perfusion-weighted magnetic resonance imaging in chest diseases” [4]. Nowadays, he is assistant professor of radiology teaching an introduction to radiology as well as problem-solving skills and communication in the master of medicine program at the faculty of medicine. He is a member of different radiological societies and reviews many radiological journals. At the University Hospitals Leuven, the development of the strategic care paths of pleural diseases, respiratory oncology, esophageal cancer, and interstitial lung disease are realized by his participation in the multidisciplinary consults. Besides co-organizing the annual high-resolution CT teaching course of the Catholic University of Leuven (KU Leuven), he is trying to optimize the conditions for realizing a diagnostic platform for thoracic imaging to incorporate different diagnostic subspecialties to better integrate treatment plans leading to more personal medicine.

**Dr. Vassiliki Pasoglou (2016)**


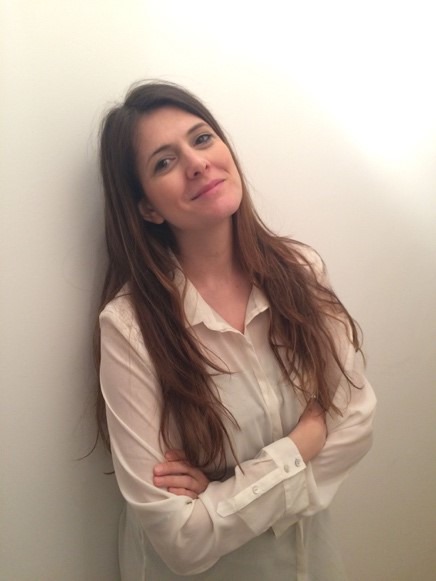


Dr. Vassiliki Pasoglou currently works as a radiologist at the Cliniques Universitaires Saint Luc in Brussels. She obtained her MD from the University of Ioannina, Greece, and following that she worked as a radiology resident at Université de Liège . After three years, she arrived at Université Catholique de Louvain in Brussels to commence her PhD thesis titled ‘’One step TNM staging of patients with high risk prostate cancer using MRI” [5]. Dr. Pasoglou was awarded the prestigious ESUI Vision Award 2015 for her work ‘’Whole-body 3D T1-weighted MR imaging in patients with prostate cancer: feasibility and evaluation in screening for metastatic disease’’. Her PhD thesis was completed in 2016, for which a summary is published in the current issue of the JBSR. She is currently engaged in research within the fields of oncological, abdominal, and whole body imaging at the Cliniques Universitaires Saint Luc.

In the first part of this PhD thesis, a critical review of the literature was effectuated concerning the use of whole body MRI (WBMRI) in oncology for the detection of bone metastasis. This review highlighted the advances of the technique over the last 15 years and designated it as a sensitive, non-irradiating, and economically credible detection tool for cancer staging. WBMRI emerged along with positron emission tomography (PET) as the methods of choice for the detection of bone metastasis. It has also been demonstrated that WBMRI can accurately detect node and visceral metastasis.

After a quick search of the literature, the reader can appreciate the major heterogeneity in acquisition protocols used in WBMRI. This important heterogeneity and the long acquisition time of WBMRI protocols—due to the addition of multiple anatomic sequences in different planes—were the basis for the first question investigated in this work: can a 3D sequence replace the multiple anatomic sequences used until today for the node and bone staging of prostate cancer (PCa)? We constructed a new 3D T1 WBMRI sequence, and we demonstrated that it can potentially replace the anatomic sequences currently used for the M and N staging of PCa.

The role of multiparametric MRI of the prostate is already established, and we demonstrated that WBMRI is a credible tool for the detection of bone and node metastasis. The second part of this work investigated the possibility to effectuate the complete TNM staging of PCa in one MRI session. We proposed the development of a WBMRI protocol, which can perform the complete T (local), N (regional), and M (distant) staging of PCa during a single MRI session in less than an hour. This all-in-one protocol is proven to be as efficient as the sum of the exams used until today for the staging of prostate cancer (MRI of the prostate for the T staging, thoraco-abdominal CT for the N staging, and bone scintigraphy for the M staging).

**Dr. Laurens De Cocker (2015)**


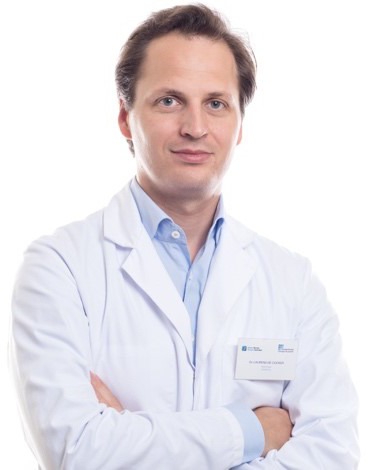


Dr. Laurens De Cocker is responsible for clinical neuroradiology and head and neck radiology in Kliniek Sint-Jan in Brussels since 2013 and is affiliated for his research activities with the University Medical Center Utrecht since 2012. He is a scientific reviewer for Europe’s leading radiology journal *European Radiology* and will serve as editor for head and neck radiology for *JBSR* from late 2016 onwards. In 2007, he earned his MD and started his radiology training in Leuven. During residency, he was awarded for his paper on MR neuroimaging of leukemia and achieved full membership of the Belgian Society of Radiology. After finishing residency in 2012, he was supported by a grant of the BSR and completed a visiting fellowship in neuroradiology and head and neck radiology in the Mount Sinai Medical Center in New York City.

Upon his return in Europe, he received a grant of the European Society of Neuroradiology (ESNR) to join the neurovascular MRI research group of Prof Dr. Jeroen Hendrikse in the University Medical Center Utrecht. In 2013, he was one of the first radiologists to obtain the European diploma in radiology during the European Congress in Vienna, and he completed the renowned AIRP (former AFIP) four-week course in Bethesda near Washington DC later that same year. In 2014, he completed the ESNR Lasjaunias cycle and acquired the European diploma in neuroradiology during the Symposium Neuroradiologicum in Istanbul. In the same year, he also served as an invited speaker for the Dutch masterclass in neuroradiology in Utrecht. In 2015, he actively participated in the European Spine Course in Antwerp. In 2016, he already hosted the joint meeting of the Belgian and Dutch Societies of Head and Neck Radiology in Brussels. Even more recently, he acted as a faculty member of the international Update in Neuro Imaging conference in Bruges.

The research of Dr. De Cocker focuses on the translation of advanced MRI of ischemic cerebrovascular diseases to routine clinical MRI [6]. He has been applying the techniques above to study the 3 P’s of cerebrovascular diseases: pipes, perfusion, and parenchyma. (*Pipes*) Application of 7T MRI with and without the administration of gadolinium for a better visualisation of the arterial vessel lumen and vessel wall. (*Perfusion*) Application of territorial ASL to study the different arterial brain perfusion territories. By (super) selective labelling of the brain-feeding arteries, territorial ASL has been allowed to image the cerebellar perfusion territories in healthy subjects, in particular the territory supplied by the PICA (posterior inferior cerebellar artery). More recently, it was shown in ischemic stroke patients that the territories of cerebral infarctions can be easily misclassified without use of a territorial imaging technique. (*Parenchyma*) Using high-resolution 7T MRI of cerebellar specimens, it was shown that cerebellar infarct cavities preferentially affect the cortex with a characteristic sparing of underlying white matter, which subsequently allowed the identification of cerebellar cortical infarct cavities as a cerebrovascular risk factor on routine MRI scans of the brain.

The results of his research have already been presented at the RSNA in Chicago in 2013, the European Stroke Congress in Nice in 2014, and the European Stroke Organisation in Glasgow in 2015 and resulted in the successful defence of his PhD thesis in Utrecht on December 10, 2015.

**Dr. Tineke De Coninck (2016)**


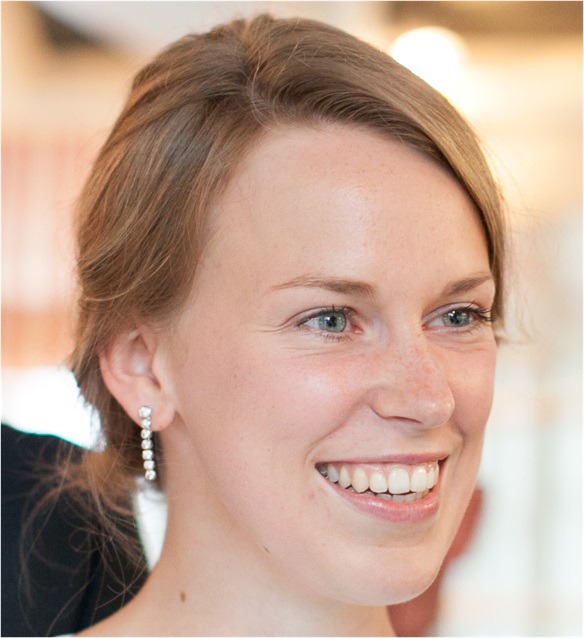


Dr. Tineke De Coninck is currently a fifth-year radiology resident at the University Hospital of Ghent. She earned her MD in 2005 with the greatest distinction at Ghent University. She started her first two years of radiology residency at AZ Groeninge Hospital at Kortrijk. Subsequently, she continued her training for six months at the Leiden University Medical Center, where she subspecialized in musculoskeletal radiology and, more specifically, in bone and soft tissue tumors. In her fourth year of residency, she became a visiting scholar at the Musculoskeletal Radiology Department of the University of California San Diego (UCSD), led by Prof Donald Resnick.

Dr. De Coninck started her research career in 2009 at the Departments of Orthopaedic Surgery and Radiology at Ghent University, mentored by Prof Koenraad Verstraete and Prof Peter Verdonk. She worked on the topic of MR imaging of meniscal substitution, presented this work at the European Congress of Radiology in Vienna in 2011, and published several A1-publications and two book chapters on this topic. In 2015, she completed her doctoral thesis titled “MR imaging of meniscal anatomy, biomechanics and substitution”, which was granted by the Belgian Society of Radiology [7].

In 2013, she carried out research on the differentiation of cartilaginous bone tumors with dynamic contrast-enhanced MRI and her paper “Dynamic contrast-enhanced MR imaging for differentiation between enchondroma and chondrosarcoma” was published in European Radiology.

In 2013, she was selected together with 10 other young radiologists worldwide for the research program Introduction to Research for International Young Academics, granted by the Radiological Society of North America (RSNA), where she received extensive training in writing and research skills.

In 2016, she engaged in several research projects with Prof Christine Chung at UCSD. She wrote a review on MR imaging of the brachial plexus, which was selected as an educational exhibit at RSNA 2016. She also continued to work on her first interest, namely, imaging the meniscus; she started a research project to image the knee meniscus and cartilage with quantitative ultrashort echo time (UTE) MRI. This project was selected as a scientific paper for oral presentation at RSNA 2016 titled “Meniscus-chondral relationship in cadaveric knees: a surrogate for meniscal mechanical axis?». Dr. De Coninck was selected by the RSNA for the RSNA Trainee Research Prize for this project.

Her most recent project was writing a review titled “Imaging the glenoid labrum and labral tears”, which was published in the October 2016 issue of *Radiographics*.
